# *ARG1* single nucleotide polymorphisms rs2781666 and rs2781665 confer risk of Type 2 diabetes mellitus

**DOI:** 10.17179/excli2018-1178

**Published:** 2018-08-27

**Authors:** Syed Fawad Ali Shah, Tahir Iqbal, Nasreen Naveed, Sumaira Akram, Muhammad Arshad Rafiq, Sabir Hussain

**Affiliations:** 1 Department of Biosciences, COMSATS University Islamabad, Park Road, Chak Shehzad, Islamabad 45550, Pakistan; 2 Department of Internal Medicine, Shifa College of Medicine, Shifa International Hospital, H-8/4, Islamabad 44000, Pakistan; 3 The Diabetic Centre, Phulgran Stop Near Toll Plaza, Murree Express Way, Islamabad 635, Pakistan

**Keywords:** arginase-1, gene, polymorphism, association, Type 2 diabetes mellitus, genotyping

## Abstract

Genetic polymorphisms mapped in the *ARG1* locus (chr6:131894344-131905472) and their functional effects on type 2 diabetes mellitus (T2DM) have not been thoroughly elucidated to date. The present study aimed to investigate an association between variant alleles at *ARG1* locus and T2DM in patients. Two *ARG1* single nucleotide polymorphisms (SNPs) were characterized in a representative sample of 500 patients with T2DM and 500 healthy volunteers. Serum lipid profile was studied by spectrophotometric analysis, while serum arginase-1 concentrations were determined by an enzyme-linked immunosorbent assay. The regions, encompassing target SNPs (rs2781665 and rs2781666), were amplified by polymerase chain reaction and genotypes were assigned by restriction digestions. A statistically significant increase was observed in the serum hs-CRP and arginase-1 levels in the subjects with T2DM than in controls (P <0.0001; for each). The variant genotypes of rs2781666 and rs2781665 were significantly associated with T2DM when compared with controls (P< 0.0001). Moreover, type 2 diabetic patients showed higher frequencies of T allele at rs2781666 and rs2781665 compared to the controls (OR = 1.7; 95 % CI=1.31-2.13; P <0.0001, and OR = 1.9; 95 % CI=1.45-2.38; P <0.0001, respectively). Haplotype T-T (chr6: 131893247-131893559) mapped at rs2781665-A/T and rs2781666-G/T displays higher frequency in the subjects when compared to the healthy ethnically-matched control samples (P <0.0001). We wish to propose, the first ever observation to our knowledge that concluding high levels of arginase-1 and the *ARG1* polymorphisms are possible causes to confer/augment the risk of T2DM in subjects originates in Pakistan.

## Introduction

Type 2 diabetes mellitus (T2DM) is a chronic disease affecting 451 million public around the world (Cho et al., 2018[5]). The International Diabetes Federation (IDF) reported in 2001 that 8 % Pakistanis are suffering from diabetes mellitus (Whiting et al., 2011[26]). T2DM is a multifaceted disorder resulting from interactions between genetic and environmental risk factors. Common risk factors like gender, age, body mass index (BMI), tobacco smoking, and hyperglycemia alone cannot sufficiently predict the incidence rate of T2DM. Therefore, investigation of additional and/or associated risk factors that may propagate the susceptibility to develop the disease is an active area of research. Studies have shown that increased levels of arginase in serum may contribute to endothelial cell dysfunction in animal models of diabetes mellitus (Bagi et al., 2013[2]; Wang et al., 2014[25]). However, genetic polymorphism in *ARG1* gene and their association with T2DM have not been explained until recently.

Arginase-1 (EC 3.5.3.1) originally detected in hepatocytes, is also expressed in various cell types including endothelial cells, vascular smooth muscle cells and macrophages (Jenkinson et al., 1996[10]; Teupser et al., 2006[23]). *ARG1* (HGNC: 663) gene may modulate individual's susceptibility to diabetic complications (Teupser et al., 2006[23]). However, there is no convincing experimental evidence to claim significant association of genetic variants mapped in *ARG1* to T2DM. Tagged SNPs in *ARG1* gene, mapped by The National Center for Biotechnology Information/SNP (NCBI/SNP) and HapMap database has been investigated to determine the association with cardiovascular disease phenotypes in European populations (Dumont et al., 2007[7]; Meroufel et al., 2009[14]). Ethnicity can strongly influence SNP frequencies and their relationship with disease development; therefore, significant milestone has been achieved in revealing the association of, aforesaid, tagged SNPs to cardiovascular phenotypes through genome-wide association studies (GWAS) (Dumont et al., 2007[7]; McCarthy and Zeggini, 2009[13]). The probable functional SNPs (rs2781665 and rs2781666) mapped in the promoter region of the *ARG1 *gene are known to be involved in genetic regulation, but in terms of association studies, *ARG1* polymorphism has not been shown in subjects with vascular complications like diabetes mellitus (DM). Here we show an association between *ARG1 *SNPs, mapped in the upstream promoter region and T2DM in patients.

## Materials and Methods

### Sample collection and processing

The research study presented here includes clinical, biochemical and genetic analyses of samples collected from patients with T2DM and their ethnicity-matched controls. The protocol was designed by following the Helsinki Declaration (2002), and was approved by the ethics review board (ERB) of COMSATS University Islamabad, Pakistan and Institutional Review and Ethics Board (IREB) of the Postgraduate Medical Institute, Peshawar, Pakistan. An informed consent was obtained from all participants of the study focused on the genetic, biochemical and, demographic data collection and analyses. One thousand samples, including 500 (mean age 44.3 ± 3.5 years) documented subjects with T2DM and 500 (mean age 44.9 ± 7.8 years) healthy ethnicity-matched controls were ascertained in this study. The standard inclusion criterion was applied based on negative blood relation between the groups. The diagnosis of T2DM was done by considering the fasting plasma glucose at ≥126 mg/dL or 2-hours plasma glucose at ≥200 mg/dL (American Diabetes Association; Expert Committee on the Diagnosis and Classification of Diabetes Mellitus, 1997[8]). Additionally, the HbA1c level >6.5 % was taken to confirm the diagnosis. Ethnicity-matched healthy control (n=500) were without the family history of diabetes mellitus and cardiovascular disease. Venous blood samples from subjects and ethnicity-matched healthy control were collected by standard procedures. The blood sample of each subject was permitted to clot at room temperature and then immediately centrifuged to get the serum for further biochemical and immunological analyses. For genetic analysis, blood samples were collected in ethylenediamine tetra acetic acid (EDTA) containing tubes to avoid the coagulation of the blood. High molecular genomic DNA was extracted from leukocytes through non-enzymatic salting out method (Sajja et al., 2014[19]).

### Biochemical analyses

Biochemical analyses were carried out using Gesan Production s.r.l-(Italy) kits for the determination of fasting blood glucose levels, total cholesterol (TC), triglycerides (TG), low-density lipoprotein (LDL), and high-density lipoprotein (HDL) from serum samples using SPECORD^®^ 50 PLUS chemistry analyzer (Analytik Jena, Germany). All assays were performed according to the manufacturer's instructions using standard protocols. HbA1c was measured using an immunoturbidimetric method (Roche Diagnostica, France). Arginase-1 levels in the serum samples were determined by using Reddot Biotech (Canada) immunoassay kit. Arginase-1 standard was run on micro test plate and the antigen concentration, in ng/mL, was determined from the standard curve, measured by AMP Diagnostics ELISA reader (Austria). High-sensitivity C-reactive protein (hs-CRP) was estimated by Tina-quant C-reactive protein (latex) high sensitive assay using a Roche/Hitachi-904 chemistry analyzer, Roche Diagnostics; Indianapolis, USA (Hussain et al., 2011[9]).

### Typing of the single nucleotide polymorphism

Polymorphism (rs2781666) mapped in *ARG1* was investigated by polymerase chain reaction (PCR) using forward primer 5'-CGG AAG GAT CTT TAA GGT GCC-3' and reverse primer 5' -CCA TGT GTC CGA TGC AGT TCT G-3'. For the genotyping of rs2781665 polymorphism, forward primer 5' AAT ATC TAG GCA ATA TGA GGA ATA CC-3' and reverse primer 5'- CCT ATT GGT GGG AAA GAA C-3' were used. The PCR reaction mixture contained 3 µL (Approximately 150 µg) of genomic DNA, 2.5 µL (125 µg) of each forward and reverse primer, 5 µL of 10X PCR buffer (0.2 M of (NH_4_)_2_SO_4_, 0.8M Tris-HCl (pH 8.8) and 0.2 % w/v of Tween-20, 4 µL of 25 mM MgCl_2_ (Solis BioDyne, Tartu, Estonia), 1 µL of the 20 mM dNTPs (Solis BioDyne, Tartu, Estonia), and 0.5 µL (2.5 U) of Taq DNA polymerase (5 U/ µL) (Solis BioDyne, Tartu, Estonia) in 31.5 µL of deionized water. PCR reactions were performed in ProFlex PCR System (Applied Biosystems, USA). PCR was carried out at: an initial denaturation step at 95 °C for 10 min, followed by 40 cycles at 95 °C for 30 s, 60 °C for 1 min, and 72 °C for 2 min, and a final extension at 72 °C for 10 min. PCR products were resolved in 2 % agarose gel through electrophoresis. T*ai*I (Thermo Scientific, USA) restriction enzyme was used for the digestion of 294 bp fragment harbouring rs2781666 SNP mapped in upstream promoter of *ARG1*. RFLP was performed in 0.2 mL tubes (Axygen, CA, USA) in a total volume of 20 µL containing 12 µL of amplified products, 2 µL of 10X G buffer (10 mM Tris-HCl, 10 mm MgCl_2_, 50 mM NaCl, and 0.1 mg/mL BSA), 0.3 µL of T*ai*I enzyme, and 5.7 µL of deionized water. M*ae*III restriction enzyme was used to investigate the 264 bp fragment harbouring rs2781665. The digested products were resolved in 3 % agarose gel through electrophoresis and visually inspected under ultraviolet light to assign genotypes, segregating in subject with T2DM, and healthy controls.

### Statistical analysis

Basic and clinical parameters of the study samples are mentioned as mean ± SD from the mean. Statistical analysis was performed with GraphPad Instat 3.05 (GraphPad Software Inc., San Diego, California). Comparison of basic and clinical variables was carried by Chi-square test and independent samples t-test. Hardy-Weinberg equilibrium was calculated by using Arlequin V3.0. Chi-square and Fisher's exact test were used to calculate the genotype and allele frequencies between the groups. Odd ratio and 95 % CI in the binary-logistic model were calculated for the assessment of association by using MedCalc Software (Acacialaan 22, 8400 Ostend Belgium). Statistical power calculation was done by using OSSE online tool (http://osse.bii.a-star.edu.sg/calculation2.php). P value < 0.05 was considered as statistically significant.

## Results

### Basic characteristics of study population

The basic and biochemical variables in all samples are presented in Table 1(Tab. 1). Age, BMI, gender, and smoking did not show any significant differences (P > 0.005) between subjects with T2DM and healthy controls. Diastolic blood pressure (P < 0.0001), systolic blood pressure (P < 0.0001), fasting blood glucose (P< 0.0001), HbA1c (P<0.0001), cholesterol, hs-CRP (P < 0.0001), and arginase-1 levels (P < 0.0001) were significantly associated with T2DM as compared to controls. Triglycerides and LDL were found to be equally distributed between the two groups (P> 0.05; Table 1(Tab. 1)). HDL levels were significantly higher in controls than subjects of T2DM (P< 0.0001; Table 1(Tab. 1)).

### Genotypes of rs2781665 and rs2781666 

The genotype and allele frequencies of *ARG1* polymorphism at rs2781666-G/T and rs2781665-A/T for cases and healthy controls are shown in Table 2(Tab. 2). The genotype distribution of rs2781666-G/T and rs2781665-A/T polymorphisms among the controls were in Hardy-Weinberg equilibrium (χ^2 ^= 1.5; P = 0.2164 and χ^2 ^= 0.0059; P = 0.9388, respectively). There was a significant difference in the distribution of genotypes for rs2781666 and rs2781665 between cases and controls (χ^2^ = 19.1; P <0.0001 and χ^2 ^= 26.3; P< 0.0001; Table 2(Tab. 2)). The rs2781666-T variant significantly associated with the increased risk of diabetes mellitus (OR = 1.7; 95 % CI=1.31-2.13; P <0.0001; T vs. G, Table 2(Tab. 2)). Similarly, variant allele T of rs2781665 showed a significant association with T2DM when compared with healthy controls (OR = 1.9; 95 % CI=1.45-2.38; P <0.0001; T vs. A, Table 2(Tab. 2)).

### Genotypic associations of arginase-1 and hs-CRP in serum

**Figure 1:**
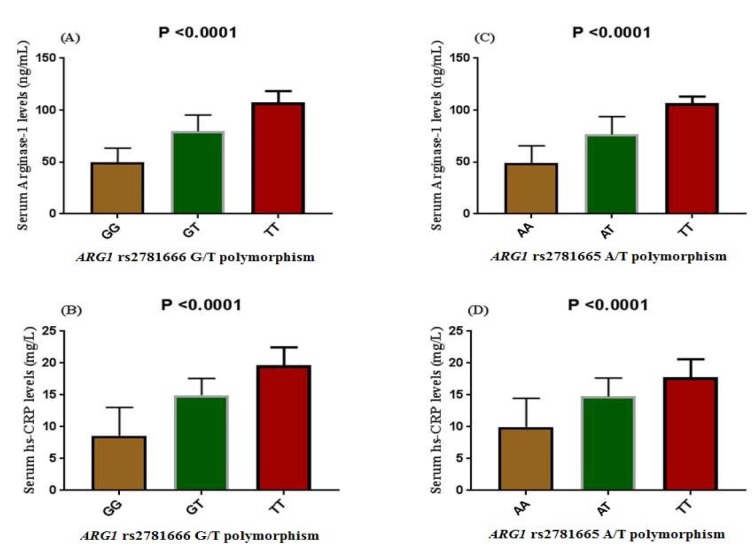
Influence of *ARG1* polymorphisms on the circulating levels of arginase-1 and hs-CRP in diabetic patients. The data represent mean ± SE. (A) Serum arginase-1 levels among the carriers of different genotype of rs2781666-G/T, P< 0.001. (B) Serum hs-CRP levels among the carriers of different genotype of rs2781666-G/T P< 0.0001. (C) Serum arginase-1 levels among the carriers of different genotype of rs2781665-A/T, P< 0.001. (D) Serum hs-CRP levels among the carriers of different genotype of rs2781665-A/T, P< 0.001. Analysis was carried out by 1-way-analyses of variance.

Subjects with T2DM showed a varying concentration of both serum arginase-1 and hs-CRP in association to rs2781666 and rs2781665 polymorphism (Figure 1(Fig. 1)). Subjects segregating variant allele T at rs2781666 had higher level of arginase-1 and hs-CRP in serum than the wild type allele G. TT genotype showed maximal arginase-1 and hs-CRP levels followed by GT and GG genotypes, respectively. A significant difference was observed in arginase-1, hs-CRP levels associated to rs2781666 mapped at *ARG1 *locus (P< 0.0001, respectively; Figure 1A and B(Fig. 1)). Similarly, an increased level of arginase-1 (Figure 1C(Fig. 1)) and hs-CRP (Figure 1D(Fig. 1)) were found in serum of the subjects segregating TT genotype at rs2781665 than wild type AA genotype (P< 0.0001). 

### Binary logistic-regression analysis

Binary logistic-regression analysis for possible association of age, BMI, hs-CRP, arginase-1, and *ARG1* polymorphism with diabetes is shown in Table 3(Tab. 3). Four variables hs-CRP (P< 0.0001), arginase-1 levels (P =0.0005), *ARG1* rs278166-G/T polymorphism (P <0.0001), and *ARG1* rs2781665-A/T polymorphism (P< 0.0001) were independently associated with T2DM. However, age and BMI were not associated with the T2DM in this study (P> 0.05; Table 3(Tab. 3)). 

### rs2781665/rs2781666 haplotype

All possible haplotype of *ARG1* variants in subjects are shown in Table 4(Tab. 4). The T-A and G-T haplotype were significantly different between the subjects and controls (P< 0.0001, respectively). Haplotype T-T of rs2781665/ rs2781666 (chr6: 131893247-131893559) illustrates a significantly higher frequency (17.80 %) in subjects with T2DM than in controls (2.6 %) (OR= 6.9; P< 0.0001: Table 4(Tab. 4)). 

Power analysis for genotypic frequencies was done by the OSSE online calculator indicates 85.6 % power for rs2781666-G/A polymorphism based on observed minor allele frequencies in cases and controls. Similarly, the observed frequency of rs2781665-A/T was 21.5 % in cases and 12.8 % in healthy individuals showed 95.5 % power in the study population.

## Discussion

Endothelial nitric oxide synthase (eNOS) is a predominant isoform, which is responsible for the oxidation of l-arginine to l-citrulline and nitric oxide (NO) in the vascular system (Romero et al., 2006[16]). However, data from several lines of evidence suggest that elevated level of arginase has a potential role in limiting l-arginine for NO production in the vascular biology (Jenkinson et al., 1996[10]). Increased activity of arginase may decrease the availability of l-arginine for nitric oxide synthase, thus reducing nitric oxide production (Beleznai et al., 2001[4]; Shemyakin et al., 2012[22]). Evidence shows that enhanced arginase may bring endothelial dysfunction in animal models of diabetes mellitus (Romero et al., 2012[17]).

In this study, we found an increased concentration of arginase-1 in patients with T2DM. The observed association of arginase-1 and SNPs at *ARG1* locus with T2DM remained significant in multivariate model when confounding variables were taken in consideration. The conclusion of this study is supported by prior observation in an experimental study showing that arginase-1 expression is up-regulated in the coronary artery of diabetic subjects (Beleznai et al., 2001[4]; Romero et al., 2008[18]). Subsequently, Alia and Munther (2017[1]) proposed that high level of arginase -1 is associated with increased risk of diabetes mellitus and cardiovascular disease. The idea was further supported by Kashyap et al. (2008[11]) after the findings of the positive correlation of circulating arginase with fasting glucose and glycosylated hemoglobin (HbA1c) levels. Similar reports (Bekpinar et al., 2011[3]; Wang et al., 2014[25]) emerged, indicating high arginase-1 is significantly associated with the diabetes mellitus in humans and rats.

Genetic variants at *ARG1* locus and their associations to T2DM have not been studied to date. Therefore, it was of great interest to investigate the association of rs2781665 and rs2781666 at *ARG1 *locus to T2DM. Interestingly, data from the current investigation revealed a significant association of aforesaid SNPs to T2DM. The variant genotypes, at both loci, have shown a significant link to T2DM. The risk of the variant alleles at rs2781665 and rs2781666 were 1.9 and 1.7-fold respectively, higher in the subjects of T2DM. We observed that T-T haplotype rs2781665/rs2781666 (chr6: 131893247-131893559) at *ARG1* locus confer the risk of T2DM. Being first ever attempt to study the genetic variants at *ARG1* locus and their associations to T2DM is incomparable and invites the scientific community to reproduce/exclude our findings by designing similar studies in different populations and /or sub-populations. However, Sediri and colleagues (2010[20]) showed concordant results of significant association between variant genotype/allele at rs2781666 and myocardial infarction. Similarly, Dumont et al. (2007[7]) showed an increased risk of developing myocardial infarction with a variant genotype of rs2781666. Conversely others have reported a non-significant association to the disease (Meroufel et al., 2009[14]). Studies showed that rs2781665-A/T is in stronger linkage disequilibrium with rs2781666-G/T and rs60389358-C/T. Minor allele T (of rs2781665) was found to be significantly associated with asthma (Litonjua et al., 2008[12]). Likewise, regulatory haplotypes generated from *ARG1* genetic polymorphisms had significantly altered the risk of asthma (Duan et al., 2011[6]). The inconsistency between our study and other studies might be the selection of disease phenotype, and/or subject ethnic backgrounds. However, findings from our study should be treated cautiously, as more in-depth studies are invited/required to confirm the novel observations, we have proposed, from another ethnic region.

In the current study, we investigated a relationship of *ARG1* polymorphism with arginase-1 levels and hs-CRP in patients. The variant genotype at rs2781666 and rs2718665 are associated with significantly higher levels of arginase-1 and hs-CRP in diabetic cases. The functional correlations between *ARG1* polymorphisms and the disease phenotype are still unknown. However, in another case-control study, the variant genotype of rs2781666 at *ARG1 *locus was significantly linked to the increased arginase activity in patients diagnosed with essential hypertension (EH). High arginase was also significantly associated to the reduced concentrations of nitrite and nitrate in subjects of EH (Shah et al., 2018[21]). The findings of the current study are consistent with previous reports showing that hyperglycemia and diabetes mellitus lead to the up-regulation of arginase-1, resulting in reduced nitric oxide-mediated dilation in human coronary arteries. Alterations in insulin signaling pathway may prone arginase up-regulation, which reduces the level of l-arginine and thereby limiting nitric oxide synthesis (Bagi et al., 2013[2]). Additionally, we showed an increased concentration of hs-CRP in T2DM associated to variant genotype at rs2718665 and rs2718666. CRP is a substantial inflammatory marker associated to increased risk of coronary artery disease in subjects with diabetes mellitus (Pfützner and Forst, 2006[15]). Positive correlation between high CRP and genetic variation in *ARG1* has been reported in a whole genome-wide association study (Vinayagamoorthy et al., 2014[24]). Data from current study showed that arginase-1 and variants of *ARG1* may be associated with increased risk of promoting an inflammatory response as evidenced by elevated CRP levels in type 2 diabetes mellitus. 

Our study has some limitation: the study is restricted to two tag SNPs of *ARG1* in Pakistani samples. The second, we could not determine the nitric oxide catabolites in this study; therefore, we intend to extend our findings to investigate the relationship between arginase and nitric oxide in diabetic patients. Therefore, replicate studies are invited to achieve a better and accurate conclusion on the baseline results provided in our study. 

In conclusion, we have demonstrated the association of arginase-1 levels and *ARG1* polymorphisms at rs2781666 and rs2781665 to T2DM in subjects originated in Pakistan. The variant genotype/minor allele T at rs2781666 and rs2781665 are contributory factors in escalating the level of arginase-1. Therefore, we wish to propose single-nucleotide polymorphisms at *ARG1 *locus are likely to be associated in generation of T2DM phenotypes in Pakistani samples.

## Acknowledgements

Authors are thankful to the Higher Education Commission, Islamabad, Pakistan, for funding under the project NO.21-523/SRGP/ R&D/HEC/201. We wish to thank all individuals for their volunteer participation in the study. The authors are thankful to the Humayoon Shafique Satti for their help in sample collection. The authors are thankful to Maahil Arshad, Victoria Park Collegiate Institute, Toronto, Canada, for proofreading the final draft of manuscript.

## Conflict of interest

No conflict of interest has been disclosed by the study investigators.

## Figures and Tables

**Table 1:**
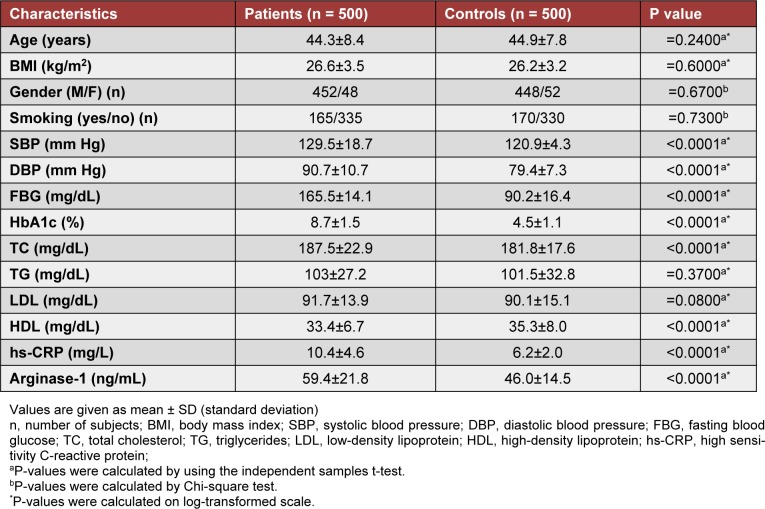
Basic and clinical characteristics of patients with type 2 diabetes mellitus and control subjects

**Table 2:**
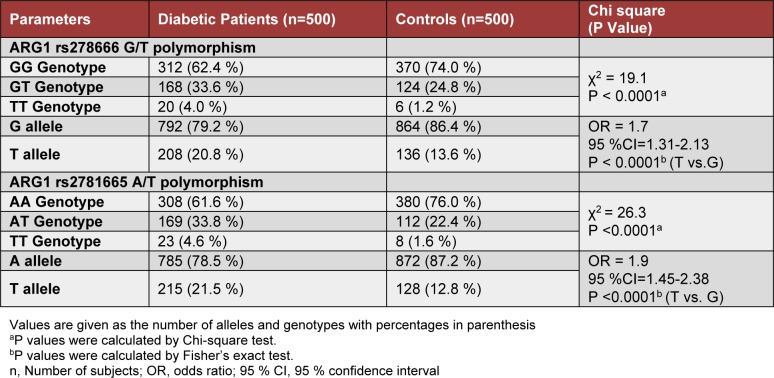
Genotype and allele distribution of ARG1 rs2781666-G/T and rs2781665-A/T polymorphisms in patients with type 2 diabetes mellitus and healthy control subjects

**Table 3:**
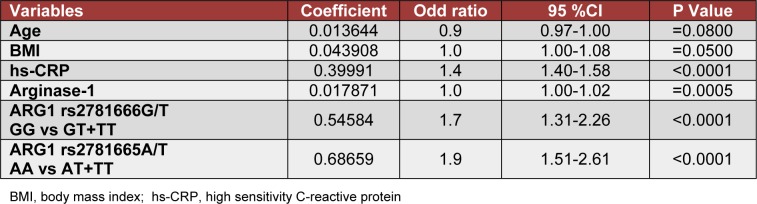
Binary logistic regression analysis in all subjects when type 2 diabetes mellitus was taken as a response variable

**Table 4:**
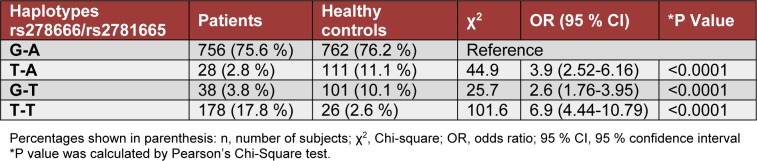
Distribution of the ARG1 haplotypes at rs2781666 and rs2781665 in type 2 diabetic patients and healthy control subjects, respectively.
